# Invited discussant comments during the UCL–Penn Global COVID Study webinar ‘Doctoral Students’ Educational Stress and Mental Health’

**DOI:** 10.14324/111.444/ucloe.100005

**Published:** 2022-12-15

**Authors:** Tara Béteille

**Affiliations:** 1 Senior Economist, The World Bank, Washington, DC, USA

**Keywords:** teachers, stress, resilience, learning, technology, Covid-19, mental health

## Abstract

This discussant commentary considers the findings presented in the UCL-Penn Global COVID Study webinar 4 ‘Doctoral Students’ Educational Stress and Mental Health’ and the research article published from the series of webinar in this journal, ‘The effects of cumulative stressful educational events on the mental health of doctoral students during the Covid-19 pandemic’. The Covid-19 pandemic has disrupted the education of hundreds of thousands of graduate students worldwide by curtailing their access to laboratories, libraries, and face-to-face interactions with peers and supervisors. This has resulted in considerable stress, given that expectations on research productivity during the period have remained unchanged. This note suggests three principles to help graduate students cope with the impact of Covid-19 on their educational journey: (1) support student resilience; (2) support student learning; and (3) support students technologically.

**About the study**

The UCL–Penn Global COVID Study^1^ launched in April 2020 is a 12-month longitudinal study of the impact of Covid-19 on social trust, mental health and physical health. In collaboration with six institutions from Italy, Singapore, the United States, China and the United Kingdom,^2^ the study looks at the short- and longer-term effects of Covid-19 on individuals’ mental health and social relationships with others. Survey data were collected at three time-points: 17 April–14 July 2020 (Wave 1), 17 October–31 January 2021 (Wave 2) and 17 April–31 July 2021 (Wave 3).

**About the webinar**

Held online between 2 June and 28 July 2021, the study group presented research data at five online webinars, as part of the UCL Global Engagement Fund sponsorship, to discuss the lessons learned and invited policy makers and other subject experts to speak on the policy relevance and implications of the study findings. The recorded comments from these discussions focusing on the policy relevance and implications of each academic article were recorded as discussant articles and are published in this journal to be read alongside the research article being discussed.

These discussant articles are reviewed by members of the Editorial Board before being published. It is hoped that these discussant articles, read alongside the academic articles, will provide more holistic understanding of the issues at hand, how findings may inform policies in the coming months and/or assist in future crisis management strategies and aid decision-making, in an open and transparent manner.

The study was pre-registered (https://osf.io/4nj3g/ on 17 May 2021) and ethical approval was obtained from the IOE (Institute of Education), UCL’s Faculty of Education and Society (University College London, UK) Ethics and Review Committee on 8 April 2020 (REC 1331)^1^.

**Linked research article**

The linked research article to this discussion article cited here has been published in *UCL Open: Environment* following open peer review and made freely available to read as an open access article. Additionally, all previous versions and peer review reports are freely available to read as open access preprint articles from the journal’s preprint server by following the below DOI link and navigating to the version history of the published research article. Readers can find more information about how peer review works in the journal at ucl.scienceopen.com.

Sideropoulos V, Midouhas E, Kokosi T, Brinkert J, Wong KK, Kambouri MA. The effects of cumulative stressful educational events on the mental health of doctoral students during the Covid-19 pandemic. *UCL Open: Environment*. 2022;(4):24. Available from: https://doi.org/10.14324/111.444/ucloe.000048.

**Recorded webinar**

This discussion article comments on the findings presented during the following webinar that has been recorded and made freely available to readers to watch on-demand.

Summer Webinar 4 – Doctoral Students’ Educational Stress and Mental Health #GlobalCOVIDStudy. Available at: https://www.youtube.com/watch?v=MIGjHVunoEI.

## Discussant comments

I would like to congratulate the authors, Theodora Kokosi, Jana Brinkert, Emily Midouhas, Keri Wong, Maria Kambouri and Vassilis Sideropoulos, as well as the entire team at the UCL–Penn Global COVID Study for this very timely and important piece of work on doctoral students’ educational stress and mental health during the coronavirus (Covid-19) pandemic. The authors have painstakingly collected data from undergraduate and graduate students on mental health at a time when Covid-19 has put a strain on the psychological resilience of many, and at a time when data collection has been especially difficult.

The importance of students being able to meet their research goals, especially during the pandemic, cannot be overstated. The role of students in important research often goes unnoticed by the outside world. For instance, the Oxford AstraZeneca vaccine – a low-cost Covid-19 vaccine, which has been a blessing in low-income countries – was the result of teamwork, with graduate students playing an essential role supporting the research in the development of trialling of the vaccine. Without their focused work, many people across the world would not have been protected from Covid-19 in time.

Prolonged and unaddressed stress makes it difficult for students to be productive. The authors’ work is especially important because it provides a glimpse of what is easily overlooked – the stresses and strains facing university students. As they show, the pandemic has made this much worse. These students are expected to produce cutting-edge and transformative research; yet their access to key facilitators of research, such as laboratories, seminar groups and face-to-face meetings has dwindled with the pandemic, while expectations from their research and financing remain unchanged.

## How can one help research students cope during Covid-19?

In this commentary, I will draw upon three principles that the World Bank has used to support teacher effectiveness during the pandemic [1]. Like doctoral students, teachers across the world have faced anxiety on multiple counts, not least because they have not been adequately supported. They have been stressed about their own health and that of their loved ones, worried about whether their jobs will end and despaired over not managing or having the technology to help students. Through all this, the expectations from teachers have remained unchanged (if not increased): to ensure students remain in school, learn and thrive.

Adapting the three principles to apply to university students:

1. Support student resilience: Covid-19 has strained the psychological reserves of all, with many worrying about the health of loved ones, financial uncertainty and anxiety about what the future holds. University students, especially those on a visa in a foreign country, as shown in the research paper, have faced similar stress. This makes it important for universities to protect student funding, on the one hand, while limiting burnout through multiple mechanisms, on the other hand. The latter include moderated social media groups to ensure regular communication among peers. Structured peer support groups are low cost, and as evidence on teachers from the Democratic Republic of Congo suggests, it can help students deal with drastic changes. Initiatives such as HealthyMinds@Work [2] can help students cultivate important aspects of well being using easy exercises based upon the latest findings in neuroscience, psychology and traditional contemplative practices. Universities can organise free webinars on mental health emphasising techniques for reducing anxiety. They can also provide students with online counselling services (or funding to access the same).
2. Support student learning: With students confined to their rooms and unable to access routine research facilities, such as laboratories, libraries and seminars, universities could encourage students to refine their non-technical skills, such as skills related to job-readiness, including job searching, writing cover letters, preparing for interviews and so forth. Universities could arrange online workshops covering these activities or support students access online courses on the same.
3. Support students technologically: Whether you are sitting in central London or northern Laos, internet services are rarely infinite or perfect [1]. We often run out of data, or the connection slows. With our work and social lives suddenly depending entirely on technology, being able to easily connect virtually and for as long as one needs is essential. Universities can support students with easy access to high-quality internet as well as offer plans for purchasing mobile phones economically. In so doing, they immediately reduce the stress generated by the uncertainty of not being able to connect with a loved one or access work.

As we live through Covid-19, thousands of university students are having to deal with tremendous amounts of uncertainty regarding their current research and personal lives as well as prospective job searches. Universities can reduce the burden students are facing through the simple mechanisms outlined above. At the same time, universities could use these principles to make long-term improvements in the support services provided to students, such that the graduate experience is more focused on students undertaking productive work and leading fulfilling lives.

## Notes

1 Wong KK, Raine A. Covid-19: Global study of social trust and mental health. Available from: https://globalcovidstudy.com [Accessed: 11 November 2021].

2 Wong KK, Raine A. Covid-19: Global social trust and mental health study. 2020. https://doi.org/10.17605/OSF.IO/FE8Q7.
